# Pathogenesis and molecular mechanisms of steroid-induced femoral head necrosis in children: a narrative review

**DOI:** 10.3389/fped.2026.1897364

**Published:** 2026-10-06

**Authors:** Fadong Gou, Lu Wu, Xingwen Bao, Wei Wang, Chen Wang, Mingxuan Gao

**Affiliations:** 1 Gansu University of Chinese Medicine, Lanzhou, China; 2 Joint Surgery, Gansu Provincial Hospital, Lanzhou, China

**Keywords:** children, genetic polymorphisms, glucocorticoids, molecular mechanisms, steroid-induced osteonecrosis of the femoral head

## Abstract

In recent years, significant progress has been made in the study of the pathological mechanisms and pathology of steroid-induced osteonecrosis of the femoral head (SONFH) in adults. However, research on the molecular mechanisms and clinical translation of SONFH in children remains relatively limited. This narrative review synthesizes recent studies and reviews the latest advances in etiology, pathogenesis, genetic susceptibility, and molecular mechanisms, with a particular focus on pediatric-specific features. Unlike adults, children's skeletons are still in the developmental stage, and their vascular architecture, metabolic characteristics, and injury repair mechanisms differ significantly from those of adults. Specifically, the open growth plate in children creates a unique vascular architecture with distinct epiphyseal and metaphyseal blood supplies, while the growth plate's elasticity provides a biomechanical buffering effect against increased intraosseous pressure. Children's bones also exhibit superior remodeling and regenerative capacity compared to adults. However, these advantages gradually diminish with age, particularly as the growth plate approaches closure during puberty. Furthermore, pediatric SONFH is a complex disease involving the combined effects of multiple genes and pathways, and the impact of genetic variations may vary depending on treatment regimens and ethnic backgrounds, further increasing the disease's complexity. In summary, pediatric SONFH has a unique molecular biological basis that is distinct from adult SONFH. Future efforts should incorporate assessment methods based on genetic susceptibility loci and biomarkers to establish a treatment model ranging from early warning to personalized intervention, thereby achieving precise prevention and treatment of steroid-induced pediatric femoral head necrosis.

## 1 Introduction

Pediatric SONFH is a condition induced by prolonged or high-dose glucocorticoids (GCs) exposure, which leads to impaired blood supply to the femoral head in children, resulting in necrosis of bone cells and bone marrow components. The current prevailing theory holds that GCs act synergistically on the pediatric skeletal system through multiple pathophysiological pathways, the predominant mechanism involves glucocorticoid-induced adipogenic differentiation of bone marrow mesenchymal stem cells, leading to fat accumulation in the bone marrow (1–3). Concurrently, elevated intraosseous pressure compresses venous sinuses, leading to microcirculatory impairment. This triggers intravascular coagulation within the intraosseous microcirculation, resulting in increased levels of prothrombotic factors such as thrombin and cholesterol, which contribute to the progression of osteonecrosis (4, 5).

The femoral head in children is in a period of growth and development. The physical growth plate separates the vascular systems of the epiphysis and metaphysis. This relatively closed blood supply pattern, lacking collateral circulation, makes it particularly sensitive to GC-induced lipid metabolism disorders, vascular embolism, and intraosseous hypertension (6). It is worth noting that the unique feature of pediatric bones lies in their strong remodeling capacity; simultaneously, the high elasticity of the growth plate can significantly buffer the pressure exerted on the internal bone structure (7). These mechanisms provide a solid foundation for bone self-repair and resistance to injury. However, this advantage gradually diminishes after puberty, as the growth plate closes.

Early genetic studies had already pointed the way toward the pathogenesis of pediatric SONFH. In recent years, with the advancement of molecular biology and genetic research, polymorphisms in genes such as PAI-1, BCL2L11, and GRIA1 have been confirmed to be closely associated with the risk of pediatric SONFH, and numerous studies have demonstrated the significant role of genetic factors in the development of the disease (8–11). These findings have not only broadened our understanding of the disease's mechanisms but also provided a theoretical basis for the early identification of high-risk children.

Pediatric SONFH is fundamentally distinct from other forms of pediatric femoral head osteonecrosis, particularly Legg–Calvé–Perthes disease (LCPD). From a pathophysiological perspective, the hallmark event in LCPD is one or more episodes of interruption of the femoral head blood supply, resulting in ischemic necrosis of the developing epiphyseal bone, bone marrow components, and the deep layer of the articular cartilage (12). In contrast, pediatric SONFH is characterized primarily by the direct effects of glucocorticoids, including inhibition of osteoblast proliferation and differentiation, promotion of osteocyte and osteoblast apoptosis, and induction of mesenchymal stem-cell differentiation toward the adipogenic lineage. Therefore, LCPD is mainly driven by dysregulated repair following an ischemic insult, whereas in pediatric SONFH, osteonecrotic injury and impaired skeletal repair may occur simultaneously.

Regarding potential etiological mechanisms, serum proteomic studies have revealed distinct molecular alterations in children with LCPD. Compared with pediatric SONFH, LCPD has been associated with increased expression of complement factor H, complement C4-B, α1-acid glycoprotein, and haptoglobin, accompanied by decreased levels of apolipoproteins E, F, and C-III (13). These findings suggest that complement–coagulation cascades and lipid regulatory networks may contribute to LCPD pathogenesis (14). However, these proteomic alterations remain associative and do not establish a causal relationship. Furthermore, it remains unclear whether these molecular changes represent initiating factors preceding ischemic injury or secondary responses following femoral head necrosis.

This article aims to summarize recent research findings on the etiology and mechanisms of pediatric SONFH, explore its similarities and differences compared with adult patients, and provide a theoretical reference for advancing the precise prevention and treatment as well as personalized diagnosis and management of this disease (Table 1).

**Table 1:** Key clinical studies on pediatric steroid-induced osteonecrosis of the femoral head.

Study	Population	Sample size	Key findings
Mattano et al. (19)	ALL	Large cohort	Alternate-week dexamethasone reduced osteonecrosis risk vs. continuous
Te Winkel et al. (20)	ALL	Prospective	Incidence 5.5%; risk factors: age >10, cumulative GC dose
Hoo et al. (16)	Chinese children with ALL	1,045	Symptomatic avascular necrosis incidence: 8.4% at 5 years
Kunstreich et al. (17)	ALL	1,143	Osteonecrosis incidence 7.1%; associated with asparaginase + GC
Yang et al. (6)	Patients with SLE	256	Max pulse dose most important risk determinant
Liu et al. (23)	Children with immune nephropathy	98	Shortest onset time: 6 months; age and dose related

ALL, acute lymphoblastic leukemia; GC, glucocorticoid; SLE, systemic lupus erythematosus.

## 2 Literature search method

### 2.1 Literature retrieval

A comprehensive literature search was conducted across multiple databases including PubMed, Scopus, Web of Science, and Embase. The search was performed from database inception through December 2025. Two independent reviewers screened titles and abstracts for relevance, with full-text review conducted for potentially eligible studies.

### 2.2 Search terms

The following search strategy was employed: (“*steroid-induced osteonecrosis*” OR “*glucocorticoid-induced osteonecrosis*” OR “*SONFH*” OR “*avascular necrosis*”) AND (“*femoral head*” OR “*hip*”) AND (“*children*” OR “*pediatric*” OR “*adolescent*”). Additional terms included “*molecular mechanism*”, “*genetic polymorphism*”, “*vascular injury*”, “*lipid metabolism*”, “*apoptosis*” and “*oxidative stress*”.

### 2.3 Inclusion criteria

Studies were included if they: (1) investigated steroid-induced osteonecrosis of the femoral head in pediatric populations; (2) reported on molecular mechanisms, genetic polymorphisms, or pathophysiological pathways; (3) were published in English; and (4) were original research articles, systematic reviews, or narrative reviews. Studies focusing exclusively on adult populations or non-steroid-induced osteonecrosis were excluded.

## 3 Epidemiology

In recent years, the global incidence of pediatric SONFH has shown a gradual upward trend (15). This condition typically progresses rapidly, with most affected children developing femoral head deformities; if not treated promptly, it may ultimately lead to severe impairment of hip function (16). Among children and adolescents, osteonecrosis is particularly common in patients receiving long-term treatment with drugs such as prednisone for acute lymphoblastic leukemia (ALL), with an incidence rate as high as 16.7% (17, 18). Among these, the femoral head is one of the most common sites for osteonecrosis in children and adolescents with ALL, accounting for approximately 35%–67% of all osteonecrosis sites (19–21). Additionally, due to the effects of long-term hormone therapy and vascular lesions, the cumulative incidence of SONFH is also high in children with systemic lupus erythematosus (SLE), reaching up to 15.7% (22). It is worth noting that the incidence of SONFH in children with ALL following hematopoietic stem cell transplantation is significantly higher than after conventional chemotherapy or radiotherapy (23). For example, in a study involving 943 pediatric cancer survivors, Gzczvova et al. (24) found that the cumulative incidence of symptomatic osteonecrosis following chemotherapy alone was 1.4%, whereas this rate rose to 6.8% among patients who underwent transplantation.

## 4 Anatomical basis of femoral head necrosis in children

The anatomical characteristics of the pediatric femoral head provide a unique biological basis for this condition. In the developing pediatric population, the blood supply to the femoral head exhibits significant anatomical distinctiveness. In prepubertal children, the epiphysis and metaphyseal vascular zones are tightly separated by the physical epiphyseal plate; their blood supply is derived from the junction of the shaft and periosteum, respectively, and ultimately converges into the terminal branches of the medial and lateral femoral circumflex arteries. The epiphysis of the pediatric femoral head relies on the terminal branches of the medial and lateral femoral circumflex arteries for blood supply and lacks effective collateral circulation; this blood supply pattern makes it extremely sensitive to microvascular embolic events (6). Additionally, due to the active metabolism of the growth plate and the unique characteristics of the blood supply system itself, the epiphysis of the femoral head is particularly sensitive to hormone-induced oxidative stress, making it more vulnerable to damage caused by ischemia (25). Furthermore, this pathological process is closely related to age, with children of different age groups exhibiting significant differences in blood supply structure and risk of injury. The reason for this is that as children grow older, the growth plate gradually ossifies, red bone marrow transforms into yellow bone marrow, and collateral circulation decreases accordingly, ultimately leading to a reduced resistance to ischemic injury (26).

Premature disruption of the blood supply often serves as a precursor to hormone-induced osteonecrosis. The root cause of this pathological process lies in the damage caused by hormones to the nutrient arteries, endothelial cells, and vascular smooth muscle cells (18). This vascular damage does not occur in isolation; rather, it involves multiple interconnected mechanisms including endothelial dysfunction, microvascular thrombosis, oxidative stress, and impaired angiogenesis (23). GC induce endothelial dysfunction by downregulating endothelial nitric oxide synthase (eNOS) expression and reducing nitric oxide (NO) production, leading to impaired vasodilation and increased vascular resistance (27, 28). Concurrently, GCs promote a pro-thrombotic state by increasing plasminogen activator inhibitor-1 (PAI-1) levels and reducing tissue plasminogen activator (tPA) activity, facilitating microvascular thrombosis (28, 29). Oxidative stress further amplifies vascular injury through Reactive Oxygen Species (ROS)-mediated damage to endothelial cell membranes and DNA (30). Additionally, GCs impair angiogenesis by suppressing vascular endothelial growth factor (VEGF) signaling and the phosphatidylinositol 3-kinase/protein kinase B (PI3K/Akt) pathway, reducing the formation of new blood vessels necessary for tissue repair (26, 30).

This vascular damage further induces blood stasis and tissue ischemia, ultimately leading to the progressive worsening of arterial lesions. Furthermore, the rapid growth of children's bones creates an imbalance between blood supply and demand, which synergizes with stress concentration in the weight-bearing zone of the femoral head, further increasing the risk of ischemia (8, 28). Concurrently, sex hormones during childhood growth directly influence skeletal remodeling, thereby affecting bone strength and quality. Furthermore, the direct toxic effects of chemotherapy on bone marrow and bone cells may also compromise skeletal integrity, with multiple factors acting in concert to contribute to osteonecrosis (27).

It is worth noting that, in both children and adults, the lateral epiphyseal artery is the primary source of blood supply to the femoral head; however, because the growth plates remain open in children, their ability to repair ischemic damage is superior to that of adults. Due to their strong remodeling capacity, children can, to a certain extent, resist bone damage caused by GCs (7). Furthermore, during early development, the growth plate provides elasticity to counteract increases in intraosseous pressure; however, after growth plate closure during puberty, increases in intraosseous pressure can only be transmitted through the epiphysis. Similarly, regenerative potential is not static; it gradually declines with age, particularly as the growth plate approaches closure (29–31). It should be noted that although this active remodeling capacity in children helps mitigate skeletal damage, the incidence of SONFH remains high in children with ALL and SLE. This may be related to the significantly higher doses of GCs administered to these patients compared to those with other conditions, or it may be due to the specific effects that the diseases themselves and their treatments have on skeletal physiology and metabolism (32, 33).

## 5 Etiology and pathophysiological mechanism

Research into the etiology of pediatric SONFH is constantly evolving, and its mechanisms are influenced by multiple factors. An early key study demonstrated a close association between pediatric SONFH and cumulative glucocorticoid dose, duration of treatment, and average daily dose. However, existing data on pediatric populations are largely limited, leading to ongoing controversy regarding the etiology of pediatric SONFH. Recent studies have further explored the association between patterns of steroid use and the risk of osteonecrosis.

However, these studies have yielded conflicting results. Kallas et al. (34), after collecting long-term follow-up data from 2,428 patients with SLE and conducting statistical analyses, identified a daily dose of 40 mg of prednisone for one consecutive month as the most significant risk factor for the development of SONFH. In contrast, GC pulse therapy did not significantly increase the risk of osteonecrosis, suggesting that cumulative steroid dose is the parameter most closely associated with GC use. However, Yang et al. (6), while observing the treatment course of pediatric systemic lupus erythematosus, found that although both the pulse dose and cumulative dose of prednisone significantly influenced the risk of osteonecrosis, the maximum pulse dose was the most important risk determinant. These contradictory findings may be attributed to differences in study populations (adult vs. pediatric), underlying diseases (SLE vs. ALL), and GC administration protocols. We propose that both cumulative dose and pulse dose contribute to SONFH risk, with their relative importance varying by clinical context.

Currently, there is no consensus on the duration threshold for GC exposure, and the critical risk periods identified in different studies vary. Some studies have found that the first three months of GC therapy increase the risk of SONFH (35). However, a study by Zizic et al. (18) indicated that the incidence of SONFH was higher 3 and 6 months after GC therapy. In the study by Liu et al. (36), the shortest time to onset of SONFH following GC treatment in 98 patients with autoimmune nephropathy was 6 months. Some reports suggest that this may be related to the affinity of different corticosteroids for their receptors and the risk of osteonecrosis (22, 37). In summary, early prevention is recommended for high-risk children 3–6 months after treatment, even in the absence of clinical symptoms.

Furthermore, comparisons of osteonecrosis incidence following treatment for various underlying conditions suggest the need to distinguish between the direct effects of corticosteroids on the underlying disease and the impact of combination therapy, highlighting the importance of multifactorial analysis. For example, children who have undergone kidney transplantation often present with chronic renal failure, calcium-phosphorus metabolism disorders, and hyperparathyroidism, suggesting that the risk of osteonecrosis may stem from the synergistic interaction of multiple factors rather than simply from corticosteroid exposure (38, 39). Early studies by Gregg et al. (38) and others suggest the need for a comprehensive assessment that takes into account the use of immunosuppressants, as well as metabolic abnormalities such as hypercoagulability and obesity. This finding has further shifted the focus of research from single-factor analysis to the study of multisystem interactions. Recent studies have confirmed that even low-dose GCs, such as 4.2 g/m^2^ prednisolone combined with methotrexate, asparaginase, and other multi-drug chemotherapy regimens, can still induce osteonecrosis, suggesting that non-steroidal drugs may produce synergistic toxic effects by inhibiting angiogenesis or interfering with bone metabolism (40). Concurrently, obesity, radiation therapy, and other factors are currently recognized as significant risk factors for SONFH. In addition to the synergistic risks posed by multiple factors, attention must also be paid to the persistent damage to the skeleton caused by the disease itself. Lilleby et al. (33) noted that the inflammatory response associated with SLE (particularly elevated levels of pro-inflammatory cytokines such as IL-1, IL-6, and TNF-α) exerts its effects not only through the side effects of corticosteroids but also by directly interfering with bone metabolism. Specifically, these inflammatory processes inhibit bone formation while accelerating bone resorption, thereby independently exerting a negative impact on bone health. Furthermore, patients with juvenile-onset SLE before skeletal maturation often achieve a peak bone mass (i.e., the maximum bone mineral reserve in a person's lifetime) that is significantly lower than that of healthy peers. More notably, even in early adulthood, these patients do not exhibit a significant “catch-up” trend in bone density. This situation indicates that their future risk of osteoporosis and even fractures is likely to remain persistently elevated (41). With the progress of numerous clinical and basic research studies, various theories regarding the etiology of pediatric SONFH have been proposed. Wang et al. (42) proposed five major pathogenic mechanisms for SONFH in their research: reduced osteogenic potential, lipid metabolism disorders, insufficient blood supply, apoptosis, and genetic polymorphisms. However, from an evidence-based medicine perspective, the duration of GC therapy remains the primary risk indicator and a major risk factor for osteonecrosis (9).

## 6 Molecular genetic mechanism

The pathogenesis of pediatric SONFH involves a complex network of interconnected molecular and cellular mechanisms. Rather than operating in isolation, these pathways form an integrated pathogenic framework in which vascular injury, metabolic disturbances, coagulation abnormalities, cell death, and genetic susceptibility interact to drive disease progression. The following sections describe these mechanisms within their pathophysiological context (Figure 1).

**Figure 1:**
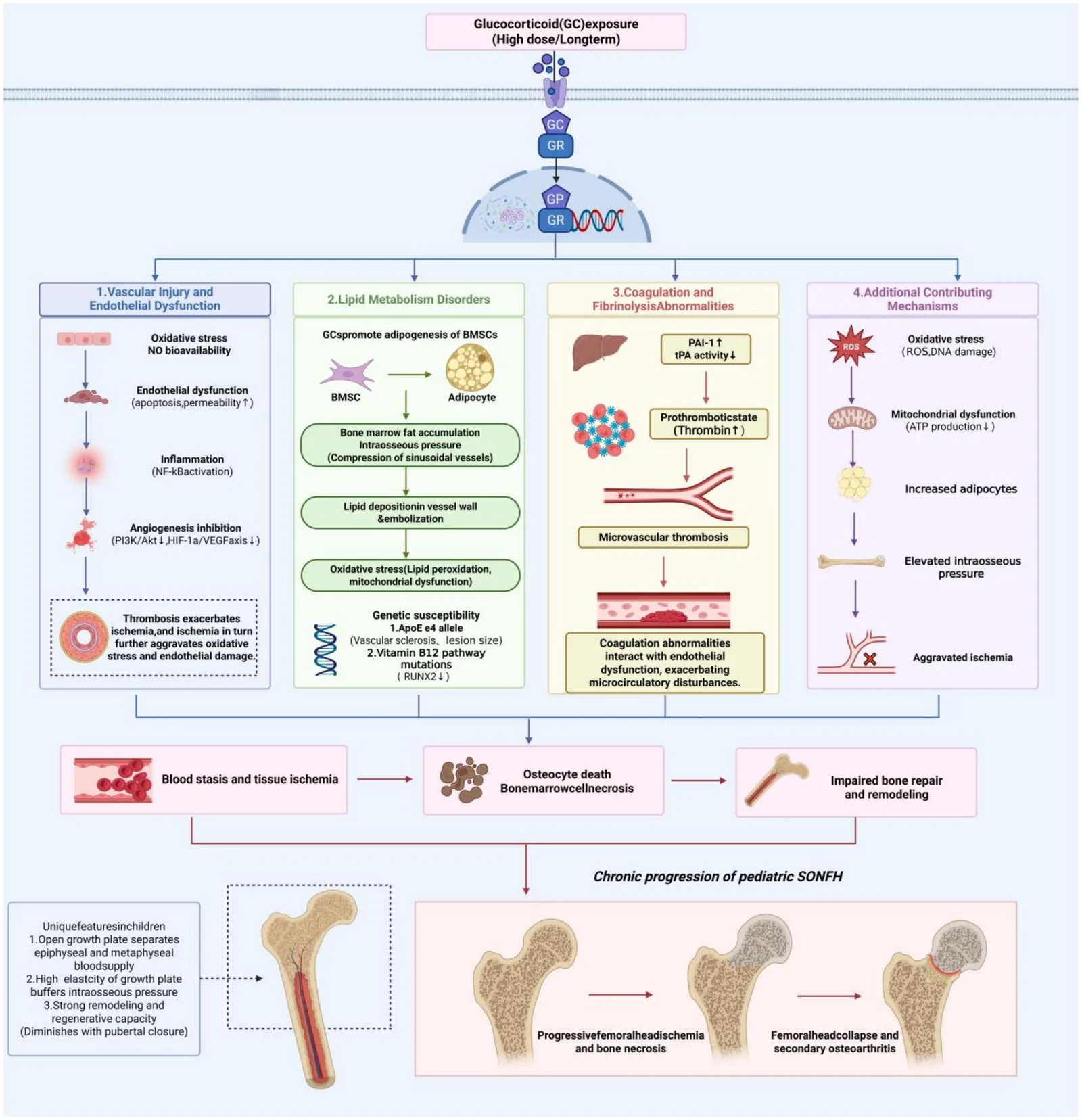
Various mechanisms of steroid-induced femoral head necrosis in children.

### 6.1 Vascular injury and endothelial dysfunction

Vascular injury represents one of the earliest and most critical events in pediatric SONFH pathogenesis. GCs directly damage the vascular endothelium by inducing oxidative stress and reducing NO bioavailability. Nuclear factor-κB (NF-κB) belongs to a family of rapidly responding inducible transcription factors. Its signaling pathway is abnormally upregulated by glucocorticoids in bone tissue, and the inflammatory response mediated by this pathway further damages vascular integrity (43, 44). Concurrently, GCs suppress the PI3K/Akt pathway in endothelial progenitor cells (EPCs), inhibiting angiogenesis and reducing the capacity for vascular repair (45, 46). The Hypoxia-Inducible Factor-1α/Vascular Endothelial Growth Factor (HIF-1α/VEGF) axis, which is essential for hypoxia-induced angiogenesis, is also impaired under GC exposure, leading to insufficient neovascularization in ischemic regions (24). These vascular changes do not occur in isolation; they establish a feed-forward loop in which endothelial dysfunction promotes thrombosis, thrombosis exacerbates ischemia, and ischemia further amplifies oxidative stress and endothelial damage.

### 6.2 Lipid metabolism disorders

GCs promote adipogenic differentiation of bone marrow mesenchymal stem cells (BMSCs) at the expense of osteogenesis through suppression of the Wnt/β-catenin signaling pathway (47–49). This leads to accumulation of bone marrow adipose tissue, which compresses sinusoidal vessels and increases intraosseous pressure. Furthermore, lipid deposition triggers oxidative stress through lipid peroxidation and mitochondrial dysfunction, creating a metabolic vicious cycle. Genetic factors also influence lipid metabolism in pediatric SONFH. Carriers of the apolipoprotein E epsilon 4 allele (ApoE ε4) among affected children exhibit more severe vascular sclerosis and larger osteonecrotic lesions (50). Mutations in vitamin B12 metabolic pathway genes elevate homocysteine levels, which impair bone repair by inhibiting RUNX2 expression (1, 51).

### 6.3 Coagulation and fibrinolysis abnormalities

A hypercoagulable state is central to the pathophysiology of steroid-induced osteonecrosis. GCs increase PAI-1 expression and reduce tPA activity, creating a pro-thrombotic environment that promotes microvascular thrombosis (52–55). In children with ALL, thromboembolism caused by microthrombi is a strong and independent predictor of osteonecrosis (50). The PAI-1 rs6092 allele frequency varies significantly across ethnic groups (30.9% in Caucasians vs. 12.5% in African Americans), contributing to differential genetic susceptibility (44). Additionally, Polymorphisms in methylenetetrahydrofolate reductase (MTHFR) gene variants and PLAT TPA25 I/D polymorphisms further modulate thrombotic risk (56, 57). These coagulation abnormalities interact with endothelial dysfunction to worsen microcirculatory impairment.

### 6.4 Cell death: apoptosis and beyond

GCs induce apoptosis of osteoblasts and bone marrow stromal cells through multiple pathways. BCL2L11 (Bim) is a key regulator of GC-induced apoptosis; its polymorphisms result in lower expression of the anti-apoptotic Bim γ isoform, leading to more pronounced osteoblast apoptosis (9, 58, 59). Dexamethasone induces BCL2L11 expression in osteoblasts in a dose- and time-dependent manner (10). Beyond apoptosis, recent evidence indicates that necroptosis and pyroptosis also contribute to SONFH. Glucocorticoids or TNF-α trigger necroptosis in bone microvascular endothelial cells (BMECs) through RIPK1/RIPK3/MLKL activation, while NLRP3 inflammasome-mediated pyroptosis releases pro-inflammatory cytokines that amplify tissue damage (29, 44, 60).

### 6.5 Oxidative stress

Oxidative stress serves as a central node connecting multiple pathological processes in SONFH. Excessive ROS production activates NF-κB and inflammasomes, promoting inflammation and osteoclast activation. ROS also directly damage osteoblasts and BMSCs, reducing the pool of cells available for bone repair (61). Kelch-like ECH-related protein 1/Nuclear factor erythroid 2-related factor 2 (Keap1-Nrf2) is the main cellular protective transcriptional axis, serving as the primary cellular defense mechanism against oxidative stress. The exposure to GC will inhibit the Keap1-Nrf2 antioxidant defense pathway, thereby further aggravating oxidative damage (62). Importantly, oxidative stress interacts bidirectionally with lipid metabolism (lipid peroxidation), vascular function (eNOS uncoupling), and cell death (mitochondrial dysfunction), forming self-amplifying pathological cycles.

### 6.6 Genetic susceptibility

Genetic polymorphisms collectively contribute to individual susceptibility to steroid-induced osteonecrosis of the femoral head (SONFH) by influencing vascular function, coagulation, bone metabolism, and cellular responses. Several genetic variants have been identified as potential modifiers of disease risk. NWD2 variants (rs2973215) have been reported to affect vascular endothelial function and angiogenic responses (10). In addition, polymorphisms in bone morphogenetic protein 7 (BMP7) (rs75161997) and PROX1 antisense RNA 1 (PROX1-AS1) (rs1891059) have been associated with osteonecrosis susceptibility in genome-wide association studies (GWAS) (63). The acid phosphatase 1 (ACP1) gene regulates osteoblast differentiation through modulation of the Src kinase signaling pathway, thereby potentially influencing skeletal remodeling and repair processes (50, 52, 64). Similarly, polymorphisms in glutamate ionotropic receptor AMPA type subunit 1 (GRIA1) have been shown to affect asparaginase sensitivity and GC response, suggesting a potential role in treatment-related osteonecrosis susceptibility among pediatric patients with acute lymphoblastic leukemia (65, 66). Furthermore, the thymidylate synthase (TS) 2R/2R genotype has been associated with an increased risk of osteonecrosis in children younger than 10 years (23). Variations in cytochrome P450 family 1 subfamily A member 2 (CYP1A2) may also influence drug metabolism and contribute to steroid-related pathological responses (31). Endothelial nitric oxide synthase (eNOS) gene variants, including the 4a allele and T-786C single nucleotide polymorphism (SNP), may reduce nitric oxide (NO) production, resulting in enhanced platelet aggregation and impaired angiogenesis (23). Additionally, MTHFR (C677T) and ATP-binding cassette subfamily B member 1 (ABCB1) (C3435T) have been implicated in increased susceptibility to SONFH following GC exposure (56).

It is worth noting that results across different studies are not entirely consistent. The GRIN3A locus did not reach statistical significance in the cohort studied by Liang et al. (67), which may be attributed to differences in racial background (allele frequencies) and sample size. This highlights the importance of considering ethnic background when interpreting pharmacogenomic findings (Figure 2, Tables 2, 3).

**Figure 2:**
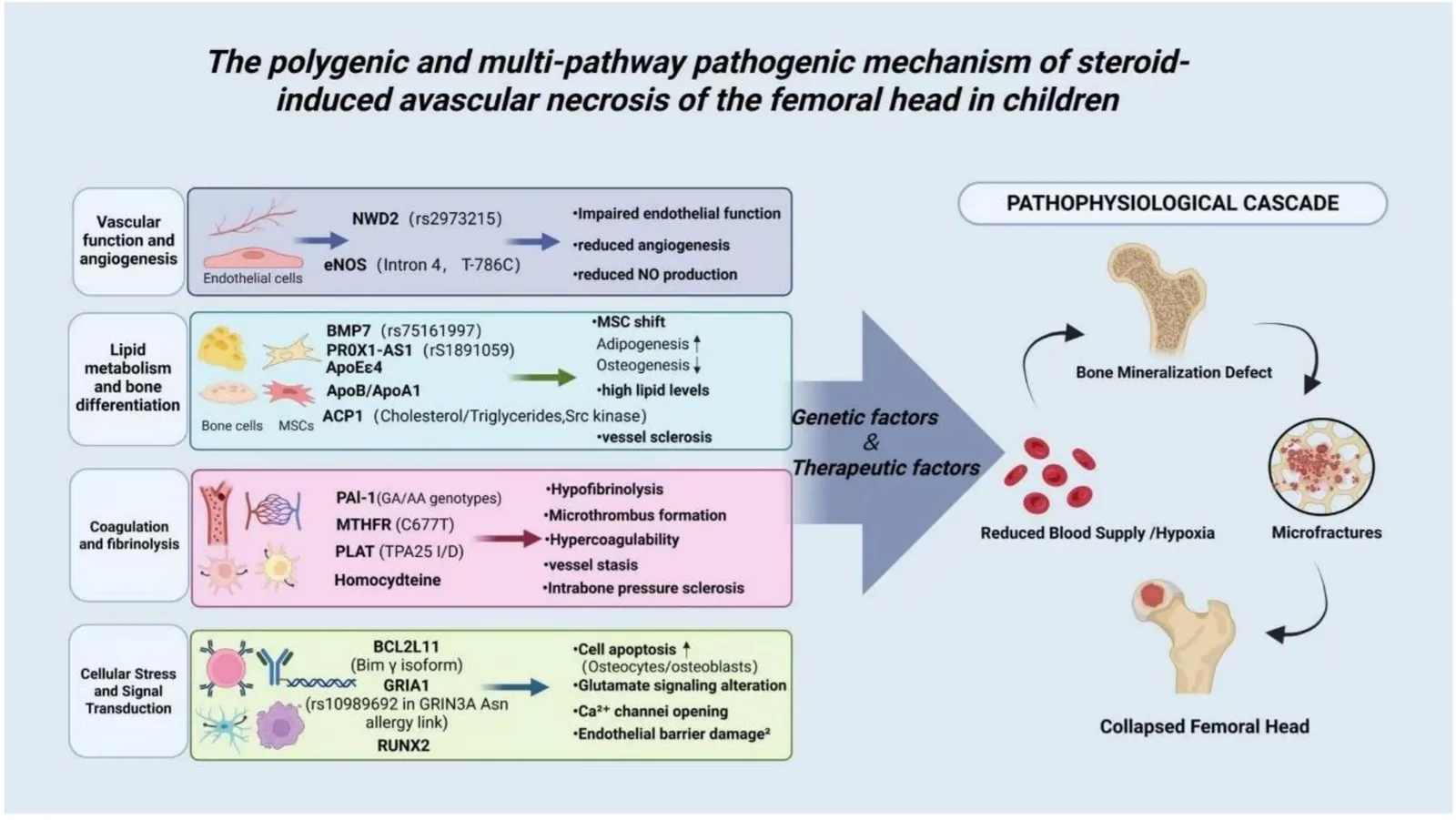
The polygenic and multi-pathway pathogenic mechanism of avascular necrosis of the femoral head in children caused by steroids.

**Table 2:** Key genetic polymorphisms and associated molecular pathways in pediatric SONFH.

Gene	Polymorphism	Pathway	Functional effect
PAI-1	rs6092	Coagulation/Fibrinolysis	Increased microthrombosis risk
BCL2L11	Multiple	Apoptosis	osteoblast apoptosis
GRIN3A	rs10989692	Glutamate signaling	Modulates asparaginase sensitivity
NWD2	rs2973215	Angiogenesis	Impaired endothelial function
BMP7	rs75161997	Osteogenesis	Altered bone-fat differentiation
ACP1	Various	Lipid metabolism/Signaling	Regulates Src kinase pathway
eNOS	4a allele, T-786C	Vascular function	Reduced NO production
MTHFR	C677T	Coagulation/Metabolism	Elevated homocysteine
TS	2R/2R	DNA synthesis	Age-dependent bone toxicity
CYP1A2	G2964A	Drug metabolism	Altered GC metabolism

PAI-1, plasminogen activator inhibitor-1; BCL2L11, BCL2-like 11 (Bim); GRIN3A, glutamate ionotropic receptor N-methyl-D-aspartate type subunit 3A; NWD2, NACHT and WD repeat domain containing 2; BMP7, bone morphogenetic protein 7; ACP1, acid phosphatase 1; eNOS, endothelial nitric oxide synthase; NO, nitric oxide; Src, Src proto-oncogene, non-receptor tyrosine kinase; MTHFR, methylenetetrahydrofolate reductase; TS, thymidylate synthase; CYP1A2, cytochrome P450 family 1 subfamily a member 2; GC, glucocorticoid.

**Table 3:** Distinguishing features of pediatric vs. adult SONFH.

Feature	Pediatric SONFH	Adult SONFH
Growth plate	Open; provides elasticity and repair capacity	Closed; no protective buffering
Blood supply	Epiphyseal and metaphyseal circulations are relatively separated by the open physis, with limited collateral circulation, making the femoral head more vulnerable to ischemia	Mainly depends on branches of the medial and lateral femoral circumflex arteries, with relatively more established collateral support
Repair capacity	High remodeling potential; can partially resist GC damage	Limited regenerative capacity
Risk factors	ALL treatment, SLE, transplantation, chemotherapy	Alcohol, trauma, systemic steroids
BMSCs alterations	BMSCs fate is influenced by both glucocorticoid injury and developmental regulation, with age-dependent characteristics	Osteogenic differentiation decreases while adipogenic differentiation increases, impairing bone repair
Oxidative stress	Due to the active metabolic process of bone tissue in children, oxidative stress may have a stronger impact on rapidly proliferating cells compared to adults.	Excessive ROS production contributes to mitochondrial dysfunction, DNA damage, and apoptosis
Inflammatory response	Inflammatory pathways may interact more closely with the age-specific immune and developmental microenvironment	Inflammasome activation and inflammatory mediators such as NLRP3, IL-1β, and IL-18 are implicated in disease progression

ALL, acute lymphoblastic leukemia; SLE, systemic lupus erythematosus; BMSCs, bone marrow mesenchymal stem cells; ROS, reactive oxygen species; GC, glucocorticoid; NLRP3, NOD-like receptor family pyrin domain containing 3; IL-1β, interleukin-1 beta; IL-18, interleukin-18.

## 7 Conclusion and future perspectives

In summary, pediatric SONFH is not simply the same pathological process observed in adults occurring in children. Its unique pathogenesis is closely related to the process of childhood growth and development. From the anatomical characteristics of the terminal branches of the femoral circumflex arteries, to the dual role of the growth plate in biomechanics and blood supply regulation, to the age-related effects observed in key genes, all of these factors indicate that preventive and therapeutic measures for pediatric SONFH must be grounded in its unique biological foundations. Although significant progress has been made in recent years regarding pediatric SONFH at the molecular level, we must acknowledge that current efforts to translate these findings into clinical practice still face significant challenges.

### 7.1 Emerging biomarkers

Future research should focus on identifying and validating novel biomarkers for early detection of SONFH. Circulating microRNAs (miR-199a-3p, miR-21), exosomal proteins, and metabolomic signatures hold promise as non-invasive indicators of early vascular injury and bone metabolism disruption. Combining genetic risk scores with clinical parameters may enhance predictive accuracy for high-risk patients.

### 7.2 Therapeutic targets

Based on the integrated pathogenic framework, multi-target therapeutic strategies should be explored. These include: (1) antioxidants (Nrf2 activators, mitochondrial protective agents) to combat oxidative stress; (2) pro-angiogenic therapies (VEGF mimetics, PI3K/Akt activators) to restore vascular function; (3) anti-thrombotic interventions tailored to genetic profiles; and (4) bone-protective agents that preserve osteoblast viability while suppressing adipogenic differentiation of BMSCs.

### 7.3 Future research directions

Large-scale, multi-center pediatric cohort studies are urgently needed to establish robust risk prediction models. Integrative approaches combining genomics, transcriptomics, proteomics, and metabolomics will provide a comprehensive understanding of individual susceptibility. Furthermore, preclinical studies using pediatric-specific animal models (e.g., growing animals with open growth plates) are essential to validate therapeutic interventions. International collaborative networks should be established to standardize diagnostic criteria, treatment protocols, and long-term follow-up protocols for pediatric SONFH.

In the future, we will need to rely on large-scale pediatric cohort data to develop predictive risk models, thereby enabling precise prevention and treatment of pediatric SONFH. With the continuous advancement of medical technology, the focus of pediatric SONFH diagnosis and treatment is shifting from symptomatic management to early prevention. By deepening our understanding of molecular genetic mechanisms and translating these insights into clinical strategies, we can significantly improve the long-term outcomes of skeletal health in children.
